# Facile fabrication of stretchable photonic Ag nanostructures by soft-contact patterning of ionic Ag solution coatings

**DOI:** 10.1515/nanoph-2021-0812

**Published:** 2022-03-17

**Authors:** Minwook Kim, Dong Kyo Oh, Jeong Dae Kim, Minsu Jeong, Hongyoon Kim, Chunghwan Jung, Jungkeun Song, Wonjun Lee, Junsuk Rho, Jong G. Ok

**Affiliations:** Department of Mechanical and Automotive Engineering, Seoul National University of Science and Technology, Seoul 01811, Republic of Korea; Department of Mechanical Engineering, Pohang University of Science and Technology (POSTECH), Pohang 37673, Republic of Korea; Etch Team, SEMES Co., Ltd., Cheonan, Chungcheongnam-do 31040, Republic of Korea; Department of Chemical Engineering, Pohang University of Science and Technology (POSTECH), Pohang 37673, Republic of Korea; POSCO-POSTECH-RIST Convergence Research Center for Flat Optics and Metaphotonics, Pohang 37673, Republic of Korea; National Institute of Nanomaterials Technology (NINT), Pohang 37673, Republic of Korea

**Keywords:** ionic Ag ink, nanoimprint lithography, polarization-sensitive color filter, soft contact lithography, stretchable nanostructure, transfer printing

## Abstract

We describe a rapid and simple method to create Ag nanostructures by using direct mechanical patterning of ionic Ag ink coating under gentle pressure, then thermal annealing to reduce the ionic Ag ink to a metallic Ag layer. The ionic liquid-phase Ag coating is easily obtained by spin-coating ionic Ag ink that has appropriate Ag concentration and can be either printed or imprinted on the desired substrate by using a soft elastomer patterning mold, then reduced to the Ag nanostructure by subsequent thermal annealing. More specifically, we present two methods: transfer printing and soft nanoimprinting. In transfer printing, the ionic Ag ink is first inked onto the elastomer mold which then contacts the target substrate to transfer the Ag nanopattern. In soft nanoimprinting, the elastomer mold conducts soft imprinting to engineer the ionic Ag ink coating to the Ag nanostructure. We systematically investigate the optimal patterning conditions by controlling the initial Ag ink concentration and the coating, printing, imprinting, and annealing conditions, to derive Ag architecture that has tunable photonic functionality. As an example, we demonstrate polarization-sensitive reflective color filters that exploit shape-tunable Ag nanostructures fabricated by soft nanoimprinting using a controllably-stretched elastomer mold.

## 1 Introduction

The silver (Ag) nanostructures have unique optical tunability and superior applicability to both laboratory and industry, and therefore have been widely used in photonic devices such as color filters [1, 2], surface-enhanced Raman spectroscopy [3], [4], [5], [6], [7], and plasmonic sensors [8], [9], [10], [11]. However, common nanofabrication protocols to create Ag nanostructures generally involve high-vacuum-assisted deposition and etching processes, which are expensive and slow, and therefore obstruct scalable and practical fabrication of the photonic and other functional devices that use Ag nanostructures [12], [13], [14]. To overcome this problem, mechanical nanopatterning processes that centrally include nanoimprint lithography (NIL) have been adopted for the facile fabrication of various Ag nanostructures [15], [16], [17], [18], [19]. NIL can readily replicate high-resolution nanopatterns by pressing the mold into a polymer resin [20], [21], [22]. Moreover, the transferred nanostructures can be tiled to cover large areas and can also be used as another mold or template [23, 24], so processes that use NIL are very favorable for high-throughput, highly reproducible, and scalable fabrication of nanostructures without resorting to high-vacuum processing and optical lithography [25], [26], [27].

An alternative strategy to fabricate Ag nanostructures uses a simple coating of solutions that include ionic Ag or dispersed Ag nanoparticles (NPs), then applies mild annealing [28], [29], [30], which is facile and scalable. Also, resin with embedded NPs has been developed for the NIL-based fabrication of photonic devices with controlled optical properties; the refractive index *n* and the extinction coefficient *k* of the NIL resins can be modulated by controlling the types and concentrations of NPs mixed with the polymeric bases [31], [32], [33], [34]. The resin that includes Ag NPs can also be engineered to form functional nanostructures by using continuous mechanical inscribing techniques [28, 35, 36]. However, the production of resin that includes well-dispersed NPs is a challenging task, and the resolution of the NIL-shaped structures obtained by Ag NPs in the resin can be limited by the volume of NPs and NP agglomerates.

Here, we demonstrate shape-stretchable Ag nanostructures that can be obtained using soft-contact patterning of a layer of ionic Ag ink. The layer of ink can be either printed or imprinted by using the polydimethylsiloxane (PDMS) molds, then readily converted to Ag nanostructures by mild thermal annealing. The initial state of the Ag-containing ink is an ionic fluid, so the Ag nanostructure can be readily printed or imprinted with controllable residual layers and in high resolutions that are not limited by the size of NPs or their agglomeration. We systematically investigate the optimal processes for patterning the ionic Ag nanostructures from resin that includes Ag NPs by controlling the initial ink concentration and its coating, printing, imprinting, and annealing conditions. The controlled stretching of flexible elastomer molds enables facile fabrication of shape-stretchable Ag nanostructures with tunable photonic characteristics. As an example, we demonstrate a polarization-sensitive reflective color filter, in which the shape-stretchable Ag nanodot pattern induces optical reflectance at a specific wavelength that shifts depending on the strain applied to the mold during the soft contact of the NIL process.

## 2 Experimental section

### 2.1 Materials

The flexible molds that bore micro- or nanoscale hexagonal or square hole arrays (nanohole molds) with various periods were fabricated by transferring hexagonal or square pillar arrays (EULITHA) in SiO_2_ substrate onto PDMS (Sylgard 184, Dow Corning) layers, then curing for 1 h at 70 °C. The surface of the PDMS nanohole mold was treated with an anti-sticking agent ((tridecafluoro-1,1,2,2-tetrahydrooctyl)trichlorosilane, Gelest) (Figure S1 in Supplementary Material), then the PDMS ‘nanopillar’ mold was prepared by transferring the nanohole pattern to another PDMS layer under the identical condition. The PDMS mold thickness was controlled to be 3–4 mm to ensure uniform printing and imprinting, and reliable stretching. An additional degassing process with a low vacuum might help to minimize defects (e.g., air bubbles) of the PDMS pattern mold.

Ionic Ag ink (TEC-CO-011, InkTec Co., Ltd.) was diluted in isopropyl alcohol (IPA, 99.5%, Daejung Chemicals & Materials Co., Ltd.) at controlled volume ratios (10–50 vol% of Ag ink in IPA) by sonication for 10 min, then filtered through a PTFE membrane (0.2 µm pore size, Advantec).

### 2.2 Pre-treatment of interfaces for successful transfer of Ag nanopatterns

The work of adhesion $\left(W_{\text{a}}\right)$ between two interfaces can be calculated by harmonic-mean equation [37, 38], given by:

(1) $$W_{\text{a}}=4\left(\frac{\gamma_{s1}^{d}\gamma_{s2}^{d}}{\gamma_{s1}^{d}+\gamma_{s2}^{d}}+\frac{\gamma_{s1}^{p}\gamma_{s2}^{p}}{\gamma_{s1}^{p}+\gamma_{s2}^{p}}\right),$$

where the subscript *s* is for the interface of solid and the superscripts *d* and *p* are for the dispersive and polar components of the surface tension γ , respectively. At this point, surface tension is determined by the geometric-mean equation [37, 38], given by:

(2) $$\left(1+\text{cos }\theta_{l}\right)\gamma_{l}=2{\left(\gamma_{s}^{d}\gamma_{l}^{d}\right)}^{\frac{1}{2}}+2{\left(\gamma_{s}^{p}\gamma_{l}^{p}\right)}^{\frac{1}{2}},$$

where θ is for the contact angle of the liquid on the solid and subscript *l* and *s* are for the liquid and solid components of the γ , respectively. By solving the simultaneous equations about the two different liquids of which know the dispersive and polar surface tensions, typically deionized (DI) water and diiodomethane, we can decide dispersive and polar surface tensions of various interfaces and calculate the $W_{\text{a}}$ between different interfaces.

The surface of PDMS molds was treated with an anti-sticking agent ((tridecafluoro-1,1,2,2-tetrahydrooctyl)trichlorosilane, Gelest) to reduce $W_{\text{a}}$ to the Ag layer. In addition, O_2_ plasma treatment was applied to the surface of the target substrate to strengthen the adhesion to the Ag layer (Figure S1 in Supplementary Material).

### 2.3 Fabrication of Ag nanostructures by soft contact-based transfer printing

The ionic Ag inks with various concentrations (i.e., 10, 20, and 30 vol%) were spin-coated on pre-cleaned general-purpose slide glass (HSU-1000412, Paul Marienfeld GmbH & Co. KG; ‘glass’ hereafter) at 100 rpm for 20 s, and then at 300 rpm for 10 s. The PDMS nanohole mold was then gently attached to the substrate that had been coated with ionic Ag ink, for the selective adsorption of the ionic Ag ink on the top surface of the nanohole mold. The ionic Ag-inked PDMS mold was carefully detached from the glass substrate, then brought into contact with the target substrate. The soft contact between the substrate and the PDMS mold was maintained for 20 s at 90 °C, under contact pressures of 0 (‘put’), 2, 5, or 10 kPa. Specifically, the pressurized force was precisely controlled on the balance and contact pressures were calculated by dividing the applied force by the uniform contacting area of soft mold. Then the mold was carefully detached from the Ag pattern-printed substrate, which was then annealed for 300 s at 180 °C to reduce the metallic Ag nanohole nanostructure.

### 2.4 Fabrication of Ag nanostructures by soft NIL

First, 50 vol% ionic Ag ink was spin-coated on the glass substrate at coating speeds of 2000, 3000, or 4000 rpm. The Ag ink-coated substrate was then soft-baked at 70 °C for 0, 1, 2, 3, or 4 min, then cooled to room temperature (RT). The PDMS nanopillar mold and the soft-baked Ag-ink-coated substrate were first adhered to each other by gently rubbing by using a roller, then subjected to NIL at gentle pressure (∼94 Pa) during baking for 5 min at 180 °C. The imprinted Ag nanohole structure was completed by demolding and cooling to RT.

To fabricate the stretchable Ag nanodot array with increased thickness as photonic structures, the PDMS nanohole mold was stretched by applying controlled strain, then fixed using a heat-resistant polyimide tape on a dummy wafer. Then 0.2 ml of undiluted ionic Ag ink was drop-cast on the glass substrate, then the stretched mold was brought into contact with the ink layer. With the contact maintained, the mold-substrate assembly was put in a low-vacuum chamber (∼0.7 bar) for 5 min to facilitate infiltration of the Ag ink into the nanohole openings in the mold, and to help minimize the residual layer in the contacted region, then subjected to NIL at 1 MPa at 110 °C for 15 min, then demolded and cooled to RT.

### 2.5 Characterization

The scanning electron microscopy (SEM) images were taken using a JEOL JSM-6700F field-emission SEM (typical operation voltage: 10 kV) after sputtering a thin Pt film with 2–3 nm thickness to prevent electron charging. Optical transmittance and reflectance measurements were obtained using a UV-VIS Spectrophotometer (UV-2600Plus, Shimadzu, Japan), using bare glass as a reference. Reflection spectra were simulated using a commercial FDTD software (Lumerical FDTD), with periodic boundary conditions along the *x* and *y* axes and a perfectly-matched layer along the *z*-axis (see the markup in Figure 5 for the coordinate), using *n* = 1.462 for the SiO_2_ substrate. Contact angles on various substrates and the Ag layer were precisely measured by the contact angle meter (Smart drop, Femtofab, Korea).

## 3 Results and discussion

**Figure 1:**
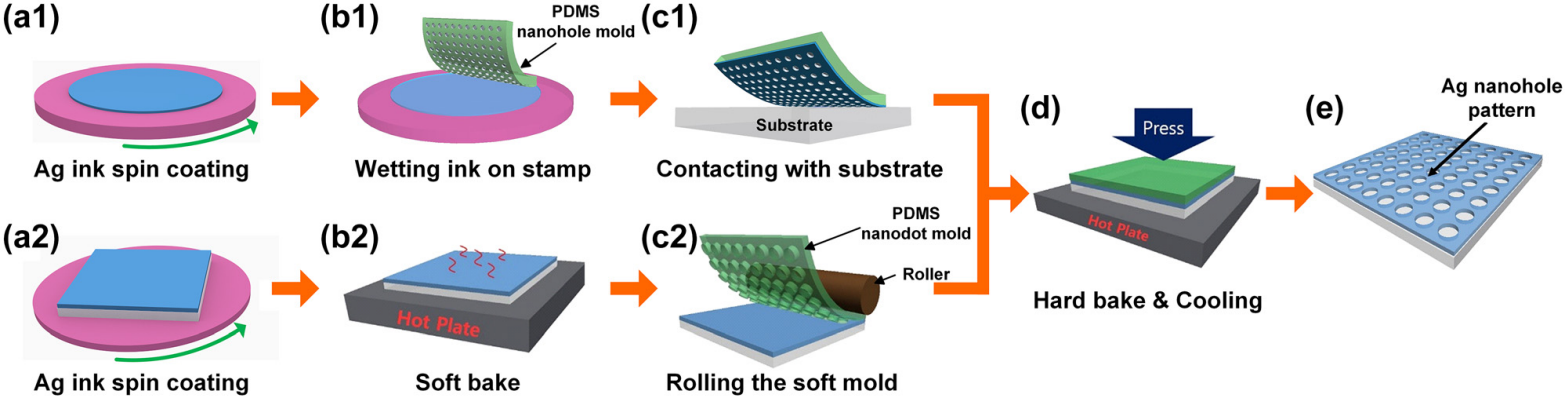
Schemes of the procedures of soft-contact patterning of Ag nanostructures in two main routes. Transfer printing of Ag nanostructures proceeds in the order of (a1) ionic Ag ink coating, (b1) wetting the PDMS mold, and (c1) contacting with the target substrate and Ag-inked PDMS mold. Soft NIL process of Ag nanostructures consists of (a2) ionic Ag ink coating on the target substrate, (b2) soft baking of the ionic Ag ink layer, and (c2) uniform contacting of the PDMS mold by using a roller. Both processes follow (d) hard baking with gentle pressure and cooling to complete (e) final Ag nanopatterns.

The proposed soft-contact mechanical patterning processes (Figure 1) use a flexible PDMS mold and an ionic Ag ink in two main steps: transfer printing and soft NIL. During transfer printing, the Ag ink concentration and the transfer contact pressure strongly influence the structural morphology and optical properties of the final Ag nanostructure. During soft NIL, the initial conditions (i.e., thickness, viscosity) of the soft-baked Ag layer have a strong effect on the subsequent NIL result, which can be adjusted by controlling Ag concentration and the coating speed of and soft-baking time (SBT) of Ag ink.

**Figure 2:**
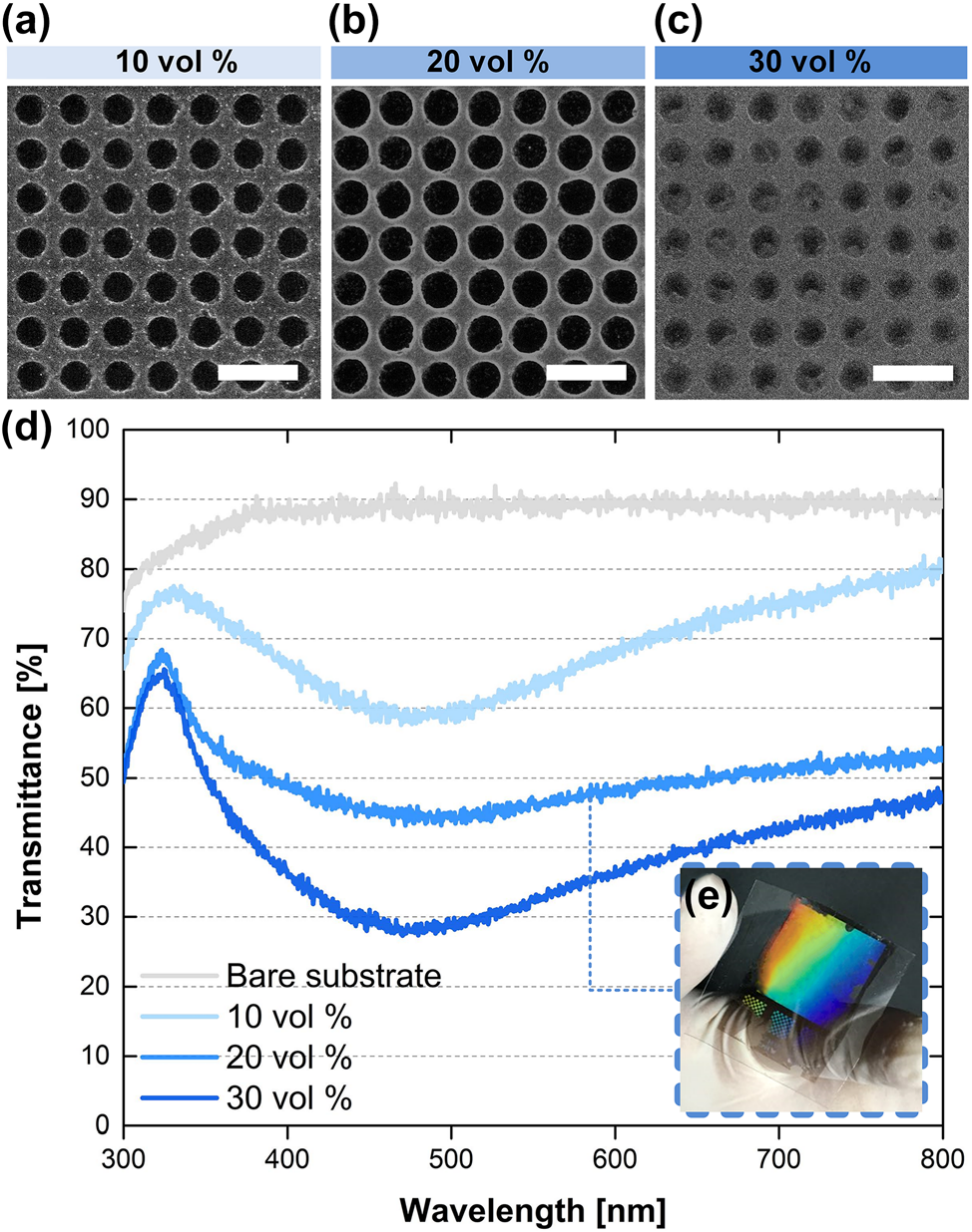
Ag nanohole patterns fabricated by transfer printing of ionic Ag ink. SEM images of Ag nanostructures fabricated by transfer printing of ionic Ag ink diluted with (a) 10, (b) 20, (c) 30 vol% in isopropyl alcohol (IPA). (d) Optical transmittance of the Ag nanohole patterns fabricated by using the ionic Ag inks with various concentrations. (e) Optical photograph of the Ag nanohole structure transfer-printed on the glass substrate by using a 20 vol% ionic Ag ink. All scale bars: 2 µm.

The transfer printing route (Figure 1a1–c1) can create Ag nanohole structures by transfer printing of an ionic Ag ink on the desired substrate and subsequent thermal annealing, by using the PDMS nanohole mold (hole diameter 800 nm, period 1.2 µm). As described in the *Experimental* section, during the transfer printing process, the Ag ink that coated the top surface of the mold was transferred to the substrate under adequate contact pressure (∼2 kPa), so essentially no residual layer remained (Figure 2a and b). Here, the ionic Ag ink concentration determines the thickness, morphology, and pattern quality of the resulting Ag layer. If the concentration of the ionic Ag ink is too low, the transferred Ag structure has insufficient Ag ions and is too porous and thin to form a continuous Ag layer that has a clean pattern profile. In contrast, if the concentration of the ionic Ag inks is too high, the excessive Ag material leaks into and fills the opening area (Figure 2c). The optimal condition to create a faithful Ag nanohole pattern with a clean opening profile was 20 vol% ionic Ag ink and contact pressure of 2 kPa (Figure 2b). This Ag nanostructure that has been transferred by soft contact can provide tunable optical transmittance depending on the ionic ink concentration (Figure 2d). The appropriate contact pressure depends on the pattern shape and scale; for instance, a 200-nm-wide Ag nanograting pattern that has a 500-nm period can be created with the most distinct profile and clean openings at 5 kPa (Figure S2 in Supplementary Material). Studies to increase the diversity of patterns will follow in the future.

**Figure 3:**
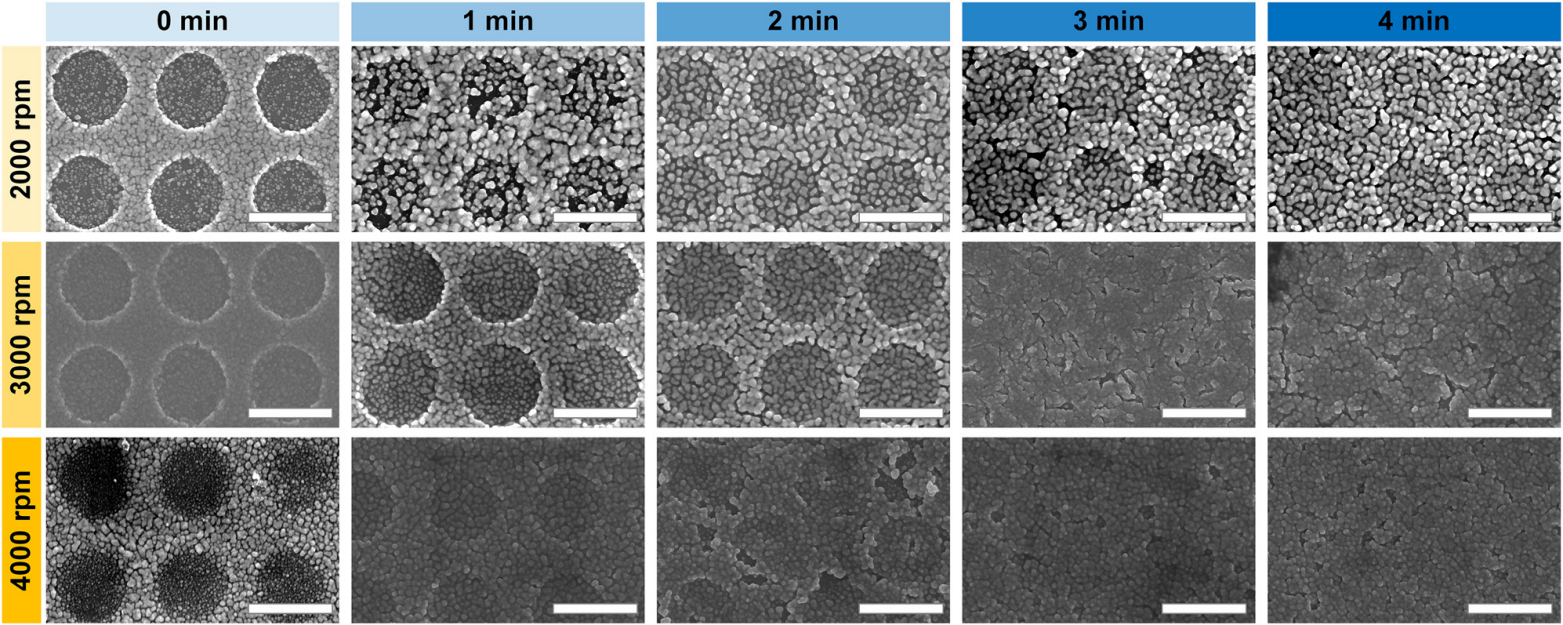
SEM images of Ag nanohole patterns fabricated by performing soft NIL onto the 50 vol% ionic Ag ink-coated substrates. The coating speeds and soft baking times for preparing the ionic Ag ink coatings were varied: 2000, 3000, and 4000 rpm, and 0, 1, 2, 3, and 4 min, respectively. All scale bars: 1 µm.

We also used soft NIL to fabricate Ag nanostructures (Figure 1a2–c2), by using the ionic Ag ink as an imprinting resin. In the soft NIL process, the PDMS nanopillar mold (pillar diameter 800 nm, pillar height 650 nm, period 1.2 µm) was applied to the soft-baked Ag layer (*Experimental* section). Here, SBT is a crucial factor for precise control of residual layer thickness as well as overall patterning quality. The use of different SBTs and coating speeds (all with 50 vol% ionic Ag ink) yielded different results. Slow spin-coating (i.e., 2000 rpm) and short SBT (i.e., approaching 0 s) are generally most favorable for the patterning using NIL (Figure 3). The definition of the nanoporous morphology in the resulting Ag nanostructure increases as SBT increases. This trend is consistent with a previous investigation, which showed that the soft-baking process initiates the reductive texturing of an ionic Ag ink layer into a nanoporous structure [29], and that the resolution of this texturing increases as the thickness of the Ag layer decreases. The residual layer thickness also increases as SBT increases (Figure S3 in the Supplementary Material). We used these findings to design an Ag nanostructure with specific photonic functionality by controlling the coating and annealing processes of the ionic Ag coating layer and by modulating the flexible mold geometry and soft NIL process condition.

**Figure 4:**
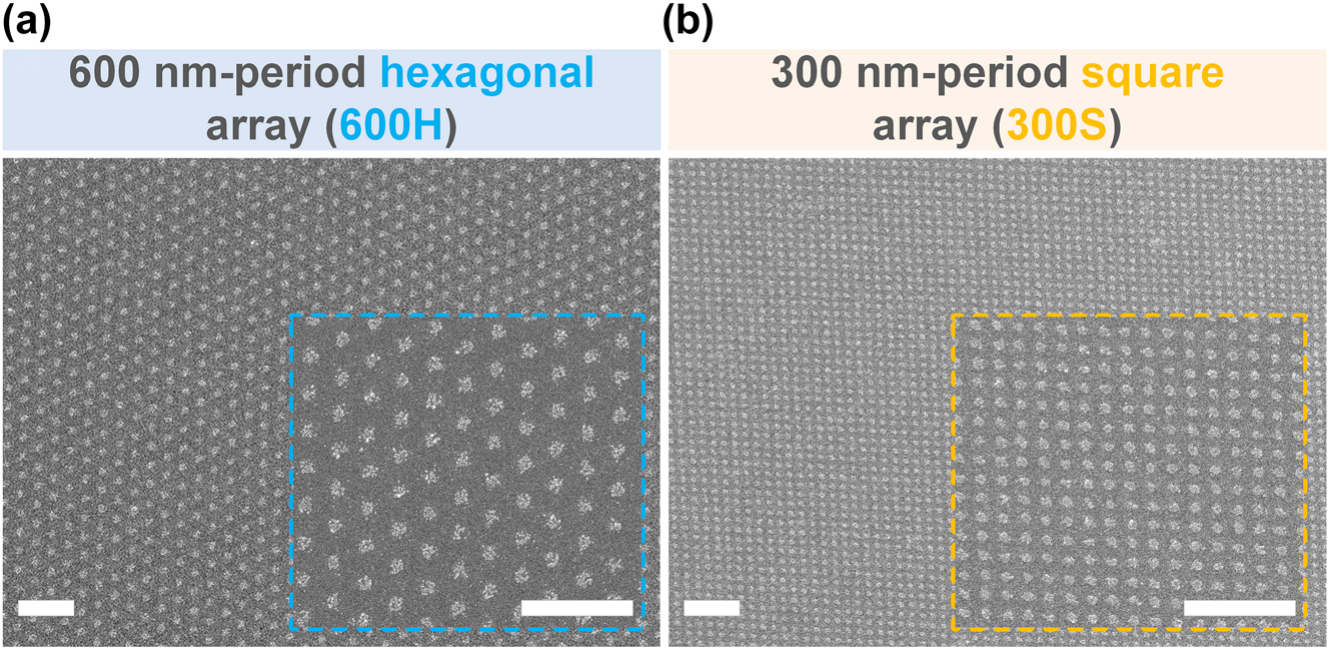
Various Ag nanodot patterns fabricated by soft NIL of the ionic Ag ink. SEM images of Ag nanodot structures with different scales and arrays: (a) 600-nm-period hexagonal array (600H) and (b) 300-nm-period square array (300S), with the corresponding inset images for enlarged views. All scale bars: 2 µm.

Here, we demonstrate one practical application: shape-stretchable Ag nanodot structures can be easily fabricated by performing soft NIL using a stretched PDMS nanohole mold (either 600-nm-period hexagonal array (600H) or 300-nm-period square array (300S), both with a 1:1 ratio of hole diameter to hole interspacing). Despite the scaling down to the nanoscale resolution, the soft NIL of the ionic Ag ink by using the modified processing conditions (*Experimental* Section) can achieve highly uniform and faithful fabrication of Ag nanostructures (Figure 4). Moving forward, the use of flexible elastomeric PDMS mold enables the fabrication of shape-tunable Ag nanostructure without the use of multiple master molds [39].

**Figure 5:**
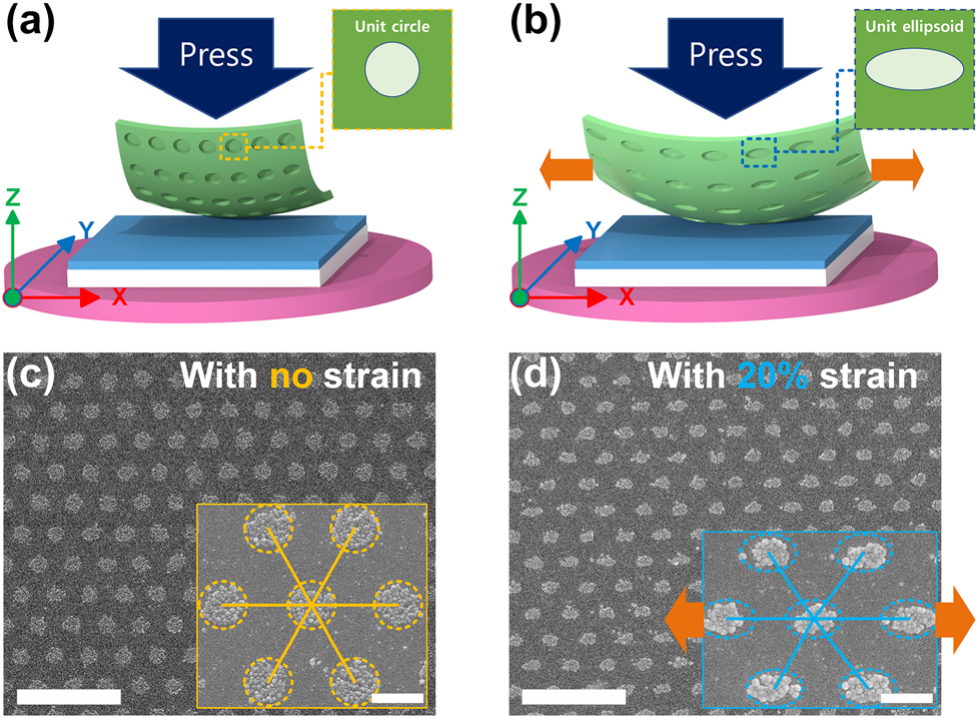
Soft NIL of stretchable Ag nanodots by using the strained PDMS mold. Graphical schematics of soft NIL of the ionic Ag ink performed by using the identical PDMS mold with (a) no strain and (b) 20% strain, respectively. SEM images of the 600H Ag nanodot patterns fabricated accordingly: (c) no strain and (d) 20% strain, respectively. The inset images show the enlarged views of corresponding structures. Scale bars: (c) and (d) 2 µm, (inset) 400 nm.

The process schemes obtained a range of NIL results (Figure 5) by using the 600H mold stretched from 0 to 20% strain. Using the unstretched 600H mold (Figure 5a), a circular Ag nanodot pattern was cleanly formed with a negligible residual layer (Figure 5c). On the contrary, by using the 600H mold stretched to 20% strain during the soft NIL process (Figure 5b), the Ag nanodot array can be readily turned into elliptical Ag patterns (Figure 5d). This practical strategy yields photonic Ag nanostructures with periods and lengths that differ between the *x*-axis and the *y*-axis (markup in Figure 5) [40]; this difference can produce linear polarization angle-dependent effective optical properties.

**Figure 6:**
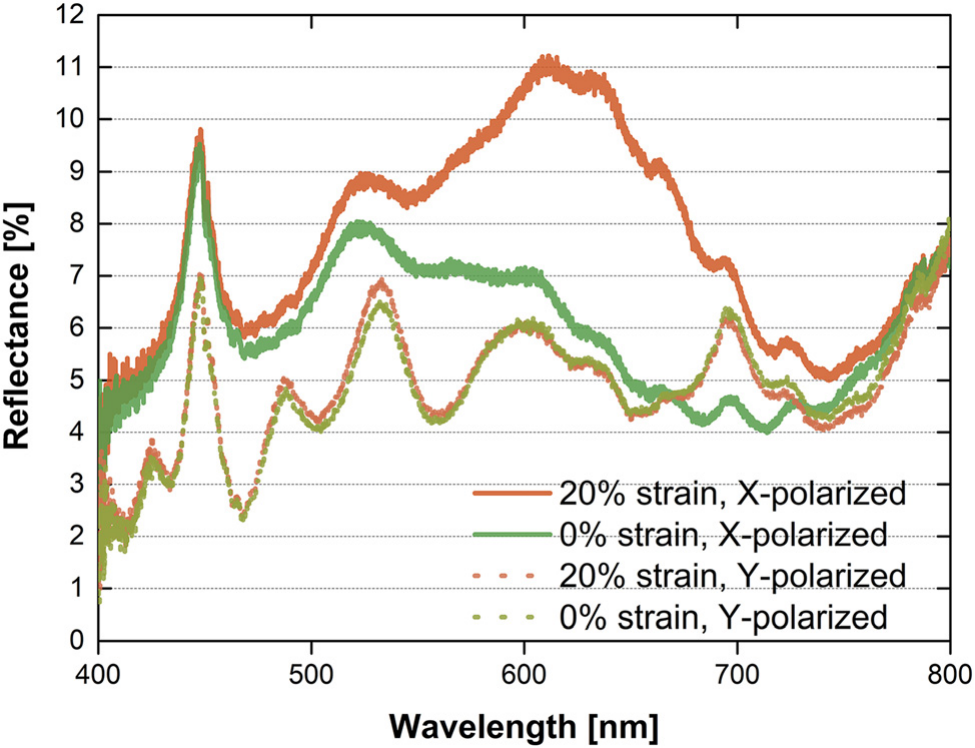
Optical reflectance of Ag nanodots fabricated by soft NIL by using the 300S PDMS nanohole mold stretched with various strains, in X- or Y-polarized light.

A simulation of the 300S pattern (Figure S4) indicates that the stretchable Ag nanodot structures can function as polarization-sensitive reflective color filters, depending on the stretch degree. Optical reflectance measurements were obtained from unstretched 300S Ag nanodot arrays and the same arrays after stretching to 20% strain, for incident light that had been polarized to 0° and 90° with respect to the stretch axis (Figure 6). Notably, the reflectance peak for the 0° linearly-polarized light was red-shifted when the Ag nanodot was stretched. On the contrary, the 90° linear polarized light generated no measurable shift of the peak position, because of the almost invariant period in the *y*-direction. Although this strategy has not been fully optimized here, it provides a pragmatic insight into applications of strain-dependent fabrication of stretchable Ag nanostructures that have tunable optical properties without the use of multiple mold prefabrication and use of high-vacuum and etching processes [41].

## 4 Conclusions

We have demonstrated two practical methods to fabricate Ag nanostructures by using soft-contact mechanical patterning of ionic Ag ink coating. *Transfer printing* can directly replicate nanopatterns with no residual layer, but contact pressure is critical to creating clean shapes of nanopatterns. In the *transfer printing* method, the Ag nanostructure could be transferred onto the desired substrate by depositing ionic Ag ink on the PDMS mold, ensuring conformal contact and adequate pressure of the mold to the substrate, then thermally annealing the ionic Ag layer. On the other hand, *soft NIL* can control residual layer thicknesses according to additional processing conditions, which induces variable optical properties of nanopatterns. In the subsequent *soft NIL* process, the PDMS mold could induce the formation of Ag nanostructures by mechanically stamping an ionic Ag coating that had been prepared under appropriate spin-coating and SBT conditions, followed by hard baking. Both *transfer printing* and *soft NIL* are complementary to one soft mold which can be stretchable for selective optical devices. To demonstrate possible practical photonic device architecture, we developed a polarization-sensitive reflective color filter by using controllably stretched PDMS mold during soft NIL of the ionic Ag coating layer. Although reusability of the PDMS mold is limited by swelling of the PDMS mold due to IPA in ionic Ag ink, the proposed strategy can be used for easy and scalable fabrication of diverse photonic devices with variable optical properties, including but not limited to the display components, transparent electrodes, metasurfaces, and plasmonic sensors [42].

## Supplementary Material

Supplementary Material
