# Supplementary material for: Facile fabrication of stretchable photonic Ag nanostructures by soft-contact patterning of ionic Ag solution coatings
Source: Nanophotonics. 2022 Mar 17;11(11):2693–700. doi: 10.1515/nanoph-2021-0812 (PMC11501842; doi:10.1515/nanoph-2021-0812)
Supplement: Supplementary file 1 — Supplementary Material [file j_nanoph-2021-0812_suppl.docx]

Supplementary Material

**Facile fabrication of stretchable photonic Ag nanostructures by soft-contact patterning of ionic Ag solution coatings**

Minwook Kim^1,†^, Dong Kyo Oh^2,†^, Jeong Dae Kim^1,†,‡^, Minsu Jeong^2^, Hongyoon Kim^2^, Chunghwan Jung^3^, Jungkeun Song^1^, Wonjun Lee^1^, Junsuk Rho^2,3,4,5,^*, and Jong G. Ok^1,^*

^1^ Department of Mechanical and Automotive Engineering, Seoul National University of Science and Technology, Seoul 01811, Republic of Korea.

^2^ Department of Mechanical Engineering, Pohang University of Science and Technology (POSTECH), Pohang 37673, Republic of Korea

^3^ Department of Chemical Engineering, Pohang University of Science and Technology (POSTECH), Pohang 37673, Republic of Korea

^4^ POSCO-POSTECH-RIST Convergence Research Center for Flat Optics and Metaphotonics, Pohang 37673, Republic of Korea

^5^ National Institute of Nanomaterials Technology (NINT), Pohang 37673, Republic of Korea

Current addresses:

^‡^ Etch Team, SEMES Co., Ltd., Cheonan, Chungcheongnam-do 31040, Republic of Korea

^†^ These authors contributed equally to this work.

*Corresponding authors:

Dr. Junsuk Rho, jsrho@postech.ac.kr, Tel. +82-54-279-2187

Dr. Jong G. Ok, jgok@seoultech.ac.kr, Tel. +82-2-970-9012


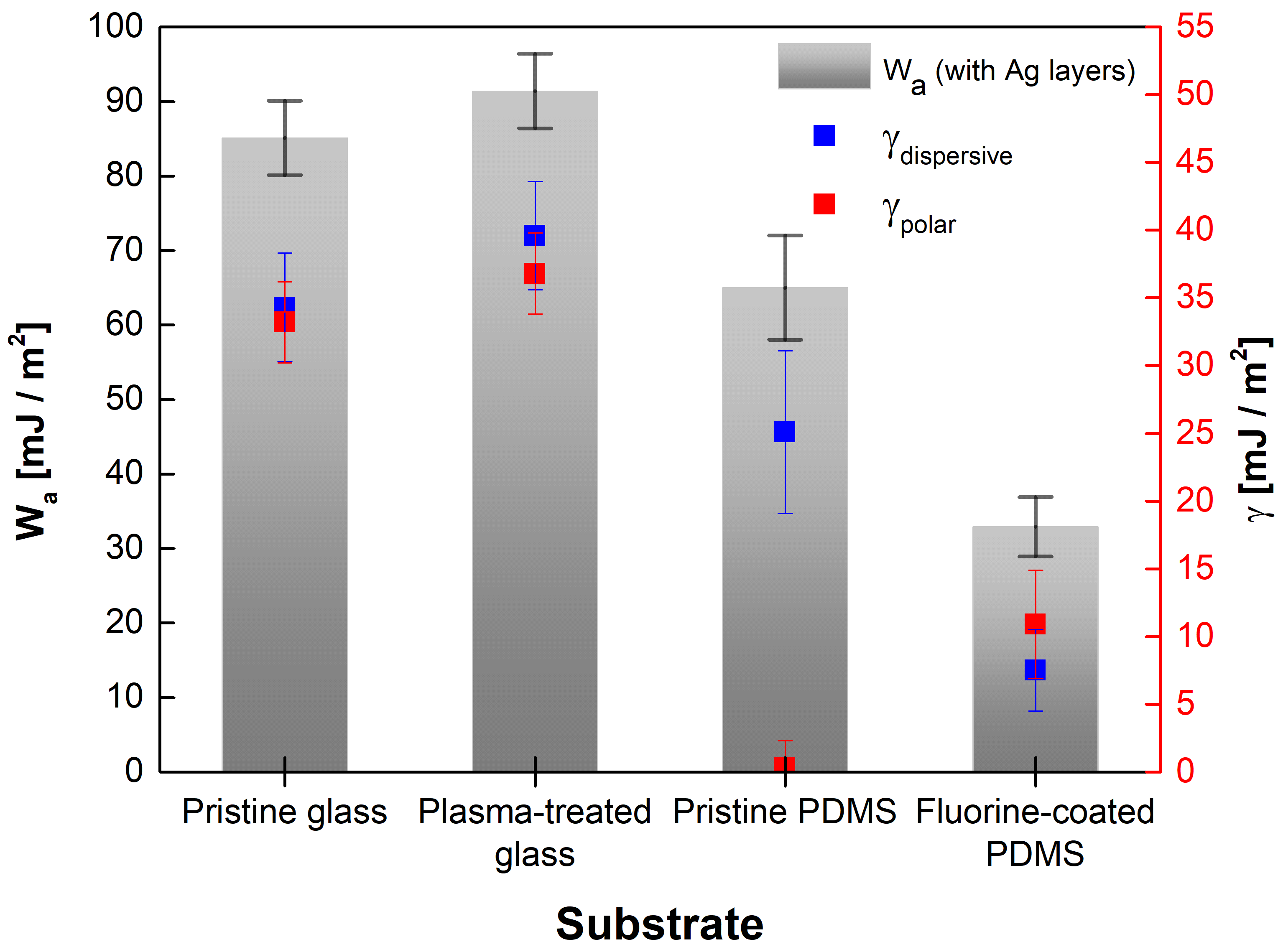


**Figure S1.** Calculation of work of adhesion (*W_a_*) values between the Ag layer and various substrates with or without surface treatment, based on dispersive and polar components of their respective surface tensions.


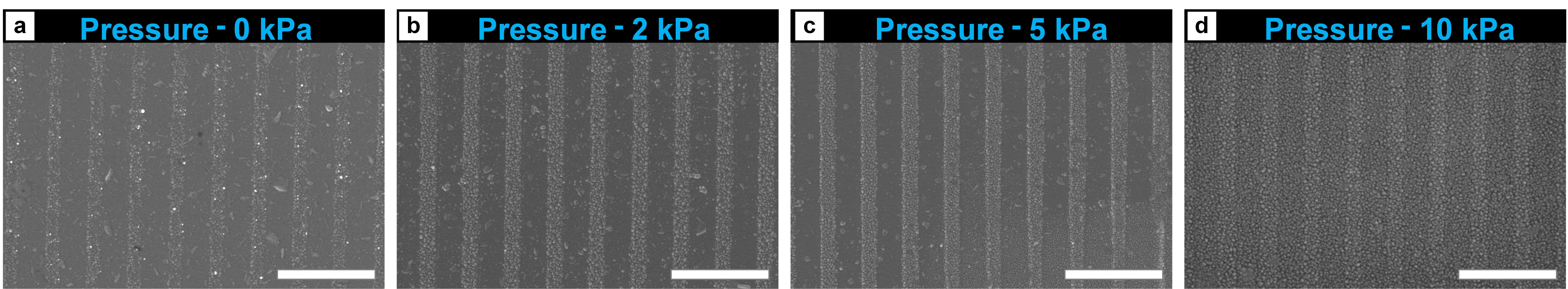


**Figure S2.** Ag nanograting patterns by transfer printing of ionic Ag ink. Scanning electron microscope (SEM) images of Ag nanostructures fabricated by transfer printing of 20 vol% ionic Ag inks at various contact pressures: (a) 0, (b) 2, (c) 5, and (d) 10 kPa. All scale bars: 1 µm.


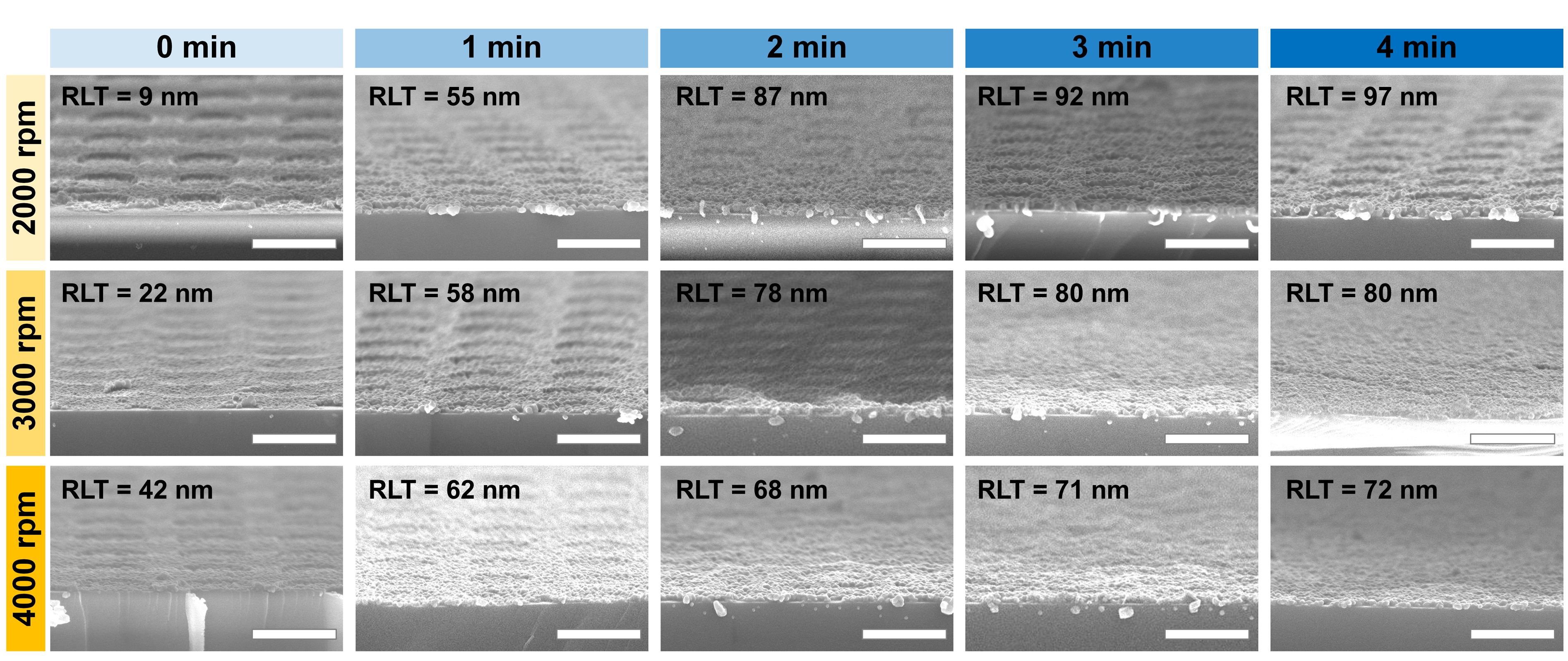


**Figure S3.** Cross-sectional SEM images and residual layer thicknesses (RLTs) of Ag nanohole patterns fabricated by soft NIL of the 50 vol% ionic Ag ink at different coating speeds of 2000, 3000, and 4000 rpm and soft baking times of 0, 1, 2, 3, and 4 minutes, respectively. All scale bars: 1 µm.


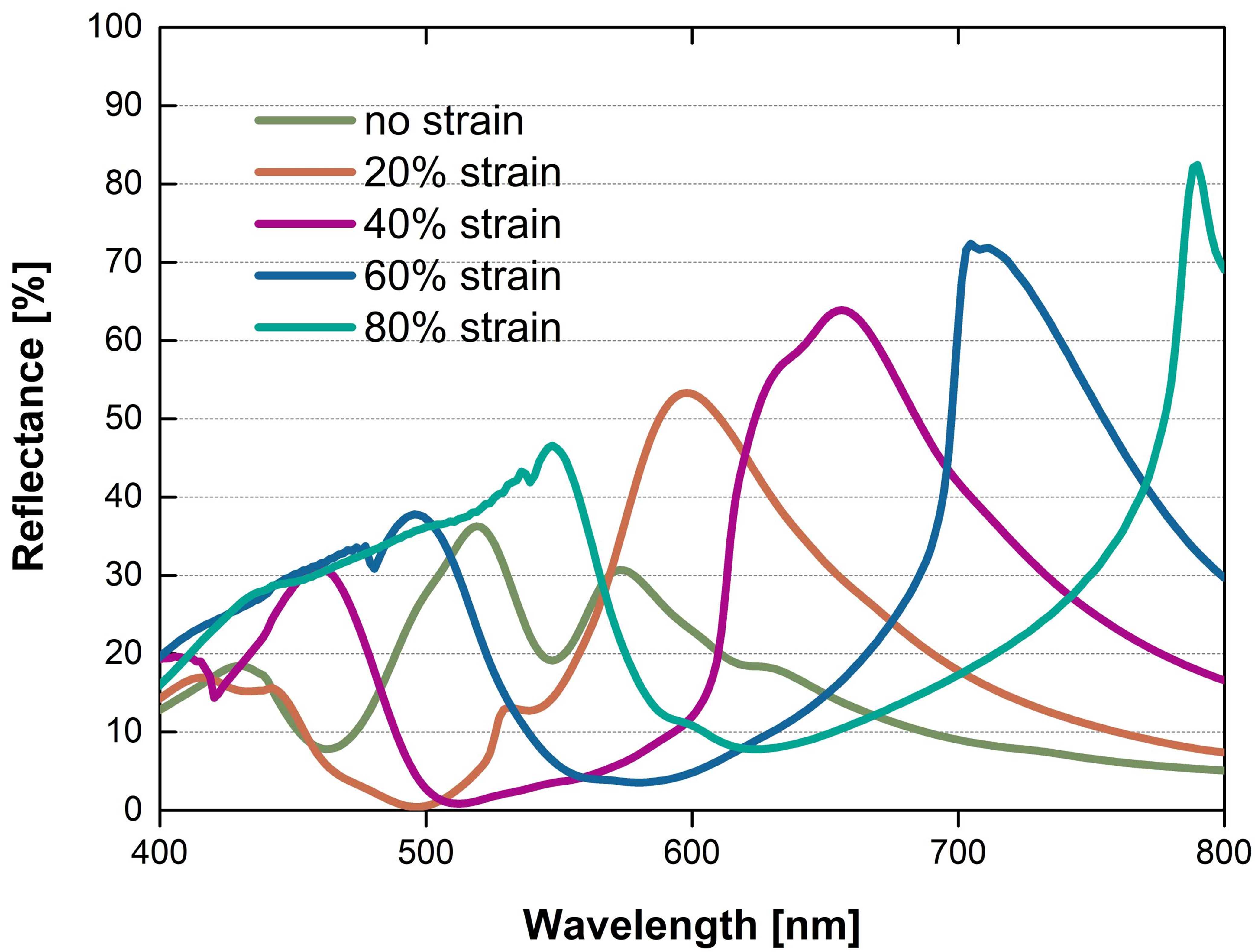


**Figure S4.** Simulated optical reflectance in X-polarized light of Ag nanodots fabricated by soft NIL with different pre-strains in the X direction of the 300 nm-period square array PDMS mold. Compared to measured reflectance, the peak of reflectance is identically shifted from 500 nm to 600 nm in simulated Ag nanostructures, which means that more deformation of the soft mold will lead to more peak shift to 800 nm.
